# Selection Route of Precursor Materials in 3D Printing Composite Filament Development for Biomedical Applications

**DOI:** 10.3390/ma16062359

**Published:** 2023-03-15

**Authors:** Aura-Cătălina Mocanu, Florin Miculescu, Andreea Elena Constantinescu, Mădălina-Andreea Pandele, Ștefan Ioan Voicu, Anișoara Cîmpean, Marian Miculescu, Andreea Mariana Negrescu

**Affiliations:** 1 Department of Metallic Materials Science, Physical Metallurgy, University Politehnica of Bucharest, 313 Splaiul Independentei, J Building, District 6, 060042 Bucharest, Romania; 2 Department of Analytical Chemistry and Environmental Engineering, University Politehnica of Bucharest, 1-7 Gh. Polizu Str., 011061 Bucharest, Romania; 3 Advanced Polymer Materials Group, University Politehnica of Bucharest, 1-7 Gh. Polizu Str., 011061 Bucharest, Romania; 4 Department of Biochemistry and Molecular Biology, University of Bucharest, 91-95 Splaiul Independentei, District 5, 050095 Bucharest, Romania

**Keywords:** materials selection, 3D printing, ABS, PLA, GNP, natural HA, in vitro testing

## Abstract

Additive manufacturing or 3D printing technologies might advance the fabrication sector of personalised biomaterials with high-tech precision. The selection of optimal precursor materials is considered the first key-step for the development of new printable filaments destined for the fabrication of products with diverse orthopaedic/dental applications. The selection route of precursor materials proposed in this study targeted two categories of materials: prime materials, for the polymeric matrix (acrylonitrile butadiene styrene (ABS), polylactic acid (PLA)); and reinforcement materials (natural hydroxyapatite (HA) and graphene nanoplatelets (GNP) of different dimensions). HA was isolated from bovine bones (HA particles size < 40 μm, <100 μm, and >125 μm) through a reproducible synthesis technology. The structural (FTIR-ATR, Raman spectroscopy), morphological (SEM), and, most importantly, in vitro (indirect and direct contact studies) features of all precursor materials were comparatively evaluated. The polymeric materials were also prepared in the form of thin plates, for an advanced cell viability assessment (direct contact studies). The overall results confirmed once again the reproducibility of the HA synthesis method. Moreover, the biological cytotoxicity assays established the safe selection of PLA as a future polymeric matrix, with GNP of grade M as a reinforcement and HA as a bioceramic. Therefore, the obtained results pinpointed these materials as optimal for future composite filament synthesis and the 3D printing of implantable structures.

## 1. Introduction

At the end of 2022, the Healthcare Additive Manufacturing (AM) Market Report estimated the global AM market size to USD 2.01 billion and forecasted its growth to USD 9.87 billion by 2030, at a CAGR (Compound Annual Growth Rate) of 22% [1]. This focused growth of the medical sector linked to the current technology trend is triggered by the aging of the population, the rise of chronic illnesses and the rapid expansion of new markets [2].

Additive manufacturing (also known as 3D printing), developed in the second half of the 20th century, is a technology that enables the construction of three-dimensional objects out of digital data (a 3D model) [2,3,4]. The recent development of AM technologies has enabled tailored patient treatment and several uses for in medicine (anatomical models, dental appliances, medical devices, pharmaceuticals, organs, tissue, and testing models) [5,6].

One of the AM group techniques, fused deposition modelling (FDM), offers a number of benefits, including a high productivity, simple material substitution, minimal operating and installation costs, automated construction, and minimal equipment requirements [2,7,8,9]. One of the main reasons why FDM is also suitable for biomedical applications is its ability to manufacture complex structures while maintaining dimensional accuracy [10]. The potential to use enhanced biomaterials combined with AM techniques is provided by ongoing technical improvements and research conducted in close regard to the medical requirements [11,12,13,14]. Numerous variables (e.g., method, material, and process parameters) affect the outcome of the final manufactured product [15,16,17,18,19].

The limited range of biomedical materials (mostly natural/synthetic polymers) that can be processed in this way is one of the many drawbacks outlined for this group of methods [2]. Acrylonitrile butadiene styrene (ABS), polylactic acid (PLA), polyvinylidene fluoride (PVDF), polycaprolactone (PCL), polyethylene terephthalate glycol (PET-G), and nylon are the most frequently employed materials in FDM for various biomedical applications (e.g., fixation devices and bone grafting), due to their biocompatible, easily biodegradable, and sterilisable features [20,21,22,23]. Apart from this, PLA has been chosen from this group due to its low toxicity and sustainable origin, and has also been endorsed by the Food and Drug Administration as a safe biomaterial [8,10,24,25]. On the other hand, the ABS resins family present outstanding dimensional stability and excellent chemical, mechanical, and heat resistance features that make them popular alternatives for the addressed field [10,26].

Designing novel composite biomaterials that work with existing hardware equipment but that can also meet the requirements for specialised applications presents the next frontier in the biomaterials sector.

Due to their potential to stimulate bone growth, minerals are intriguing candidates for bone regeneration and can be mixed with polymeric materials to boost the bioactivity [8]. Because of their similarity to the mineral component of the human bone, calcium phosphates (CaPs) were commonly explored [27,28], especially hydroxyapatite (HA) and β-tricalcium phosphate (β-TCP) [29,30,31]. Hydroxyapatite is currently recognised as the most common biomedical material, due to its excellent biocompatibility, bioactivity, and osteoconductive behaviour [32,33,34]. In general, when combined with other materials, CaPs increased the bioactivity of the developed products and had the potential to induce osteoinductive qualities; however, when used alone, they suffer in terms of fragility (one of their consistently encountered drawbacks) [12,35].

Graphene-based materials have recently attracted a significant interest in the biomedical field (e.g., as reinforcement components, due to their capacity to increase mechanical resistance, elasticity, and flexibility) [36,37]. Recently, studies have reported the possibility to incorporate such components into a ceramic or polymeric matrix [38,39,40]; however, it must be taken into account that the chemical, morphological, structural, architectural, and dimensional characteristics of graphene materials control the biological response of the products (cells viability is primarily size-dependent and better supported by materials with lower overall surface areas) [41,42].

From this perspective, the most important aspect of this study is that even though several studies have been published over the years on the development of new feedstock materials for 3D printing techniques, none of them investigated a range of potential precursors. This would guide researchers to choose the most adequate ones for their specific applications (e.g., selection by type, concentration, appropriate shape, or dimension) [4,10]. Therefore, the main scope of this article is to offer future researchers a platform for selecting the polymeric matrix and reinforcement materials for the development of 3D printing composite filaments, based on physico-chemical and biological features.

Starting from the above-mentioned considerations, here, the selection route of precursor materials is a key-factor and the first logical step to be addressed when envisioning the long-term development of composite materials for biomedical applications. In this regard, our pilot study mentioned in ref. [43] has demonstrated that PLA/HA combination alone can increase the mechanical resistance of the products by enhancing compression and elasticity resistance.

Several key points related to the selection route of optimal precursor materials will be further outlined and clarified for the first time in this study: (i) the type of polymeric matrix (of natural (PLA) or synthetic (ABS) origin) and (ii) the type of graphene-based materials (graphene nanoplatelets—GNP) of different dimensions. The reproducibility of bovine bone-derived HA [44,45] will be tested again for a positive assurance of the results. Herein, programmed experimental investigations are conducted targeting the comparative delineation of the structural (FT-IR, Raman), morphological (SEM), and in vitro cytocompatibility (indirect and direct contact studies) features of the precursor materials (prime (ABS, PLA) and reinforcement (HA, GNP) materials). This study also attempts to minimize material waste and the number of required test samples, which are forecasted during future dedicated research.

## 2. Materials and Methods

### 2.1. Sample Preparation

The precursor materials selected and investigated in this study were divided into two categories: prime materials and reinforcement materials.

The ***prime materials***, acquired from Merck KGaA (Darmstadt, Germany), refer to the chosen polymeric materials, i.e., polylactic acid (PLA) (physical shape: granules with diameters of 3 ± 0.5 mm and natural colour) and acrylonitrile butadiene styrene (ABS) (physical shape: printing filament with a diameter of 2.85 mm). They were further prepared and investigated as received, that is, without any chemical or physical treatment. Due to the in vitro analysis protocol requirements for direct contact studies (as presented in Section 2.2.4 (iv)), we also prepared polymer-based samples in the shape of plates. The fabrication of PLA and ABS plates required the following steps: (1) the thermal homogenisation of approx. 40 g of each polymer (weighed on a calibrated four decimal analytical balance (Kern & Sohn GmbH, Balingen, Germany)) through continuous stirring on a magnetic stirrer hob, up to the melting stage, at a constant temperature (~200 °C); (2) the flattening of the resulting viscous slurry between two ceramic plates (15 × 15 cm) until a thin lamella of 1 ± 0.05 mm thickness (in order to avoid material waste) was obtained; and (3) cutting the lamella into 10 × 10 mm plates. Samples were deposited in sterile Petri dishes and denominated as *new*—initial polymeric granule/filament and *plate*—prepared polymeric lamellas.

The ***reinforcement materials*** themselves were split into two categories: graphene nanoplatelets (GNP/graphene oxide) and natural HA. Two types of xGnP^®^ (XG Sciences Inc., Lansing, MI, USA) graphene nanoplatelets were acquired: grade C (physical shape: plates with a thickness of <2 nm and a diameter of <2 μm); and grade M (physical shape: plates with a thickness of ~7 nm and a diameter of 25 μm). Natural HA was synthesised by the conversion of bovine bones following a well-established and previously reported procedure [16,44,46]. First, the bovine bone tissue was processed to remove organic components (boiling in water for 2 h, removal of soft tissue residues by mechanical processes, and heat treatment at 500 °C for 2 h in an electric oven). The resulting intermediary products were then air sintered at 1200 °C for 6 h. The HA powders were obtained by ball mill grinding carried out for 2 h at 450 rpm, followed bygranulometric sorting with standardised sieves (mesh sizes of 200 μm → 40 μm (Retsch GmbH, Haan, Germany)) [47]. Three size sorts were chosen (<40 µm, <100 µm, >125 µm) for a reproducible overview of our previous work [43] and were deposited in sterile Petri dishes. Samples were denominated according to the sort size mentioned above.

### 2.2. Sample Characterisation

#### 2.2.1. FTIR-ATR Spectroscopy Measurements

The chemical structure of precursor materials was investigated by Fourier transform infrared (FTIR) spectroscopy in attenuated total reflectance (ATR) mode. The FTIR-ATR spectra were collected on a Bruker VERTEX 70 spectrometer (Bruker, Billerica, MA, USA) using 32 scans for each sample acquired at a resolution of 4 cm^−1^ in the 4000–600 cm^−1^ region.

#### 2.2.2. Raman Spectroscopy Measurements

The Raman spectra of the samples were registered on a DXR Raman Microscope (Thermo Fischer, Waltham, MA, USA) using a 532 nm laser line and 10 scans/sample in the 3200–100 cm^−1^ region. The laser beam was focused with the 10× magnification objective of the microscope.

#### 2.2.3. Morphological Evaluation

The morphological evaluation of all precursor materials was performed by scanning electron microscopy (SEM) with a Philips XL 30 ESEM TMP microscope (FEI/Phillips, Hillsboro, OR, USA). Micrograph acquisition was carried out in five randomly chosen areas at an acceleration voltage of 25 kV and a working distance of 10 mm [48].

#### 2.2.4. Biocompatibility Experiments

The biological studies carried out during this stage aimed to investigate the in vitro biocompatibility of the precursor materials through indirect contact (*i*–*iii*) and direct contact (*iv*) studies, as presented below.

For the studies performed through indirect contact, which required extraction media preparation, the materials were maintained at 37 °C for 24 h, in Dulbecco’s modified Eagle medium (DMEM, Sigma-Aldrich Co., St. Louis, MO, USA) culture without foetal bovine serum—FBS (Gibco (Life Technologies Corporation, Grand Island, NY, USA)), according to ISO 10993-5:2009 standards (material weight/medium volume ratio of 0.2 g/mL) [49]. After 24 h, the extraction media were harvested, filtered, and used to evaluate the viability of MC3T3-E1 (ATCC^®^, CRL-2593^TM^) pre-osteoblasts (cultured in the corresponding extraction media supplemented with 10% FBS). Thus, the MC3T3-E1 cells were seeded at a cell density of 8.000 cells/cm^2^ in the serum containing the extraction medium and were maintained in culture at 37 °C for 24 h and 72 h (atmosphere of 5% CO_2_). Cells cultured in the growth medium (DMEM + 10% FBS) supplemented with dimethyl sulfoxide (DMSO) (Sigma-Aldrich Co., St. Louis, MO, USA) at a concentration of 10%, a compound considered cytotoxic, served as a positive control for cytotoxicity; cells incubated in the standard growth medium were used as a cytotoxicity negative control. For each of the analysed samples, all cell culture-based investigations were conducted in triplicate.

After the experimental periods, the cells seeded in the corresponding extraction media were investigated in terms of the following.

(*i*) Cell viability using the Live/Dead qualitative assay

Cell viability was assessed using the LIVE/DEAD viability/cytotoxicity kit (Molecular Probes, L-3224, Eugene, OR, USA), which is based on the fluorescent labelling of cells. Thus, after maintaining the cells in the extraction media for 24 h and 72 h, the cell monolayer was washed twice with serum-free medium and stained with a solution of 2 µM calcein AM (acetoxymethyl) and 10 µM ethidium homodimer-1 (EthD-1) for 10 min, in the dark, in order to quantify the cell viability. Following labelling, living cells emit green fluorescence due to the hydrolysis of calcein AM ester to calcein, while dead cells emit red fluorescence due to the binding of ethidium homodimer to DNA in the case of cells with a compromised nuclear membrane. The labelled cells were visualised using an inverted microscope equipped with epifluorescence (Olympus IX71, Olympus, Tokyo, Japan) and the representative fields were captured using the imaging software cellSense Dimension Version 4.1.

(*ii*) Cell proliferation through the quantitative MTT assay

The next stage of the experimental approach targeted the capacity of the analysed extraction media to support the proliferation of the MC3T3-E1 murine pre-osteoblasts at 24 h and 72 h, respectively. Thus, in order to quantify the number of metabolically viable active cells, the MTT assay was performed (based on the reduction of a tetrazolium salt MTT [3-(4,5-dimethylthiazol-2-yl)-2,5-diphenyltetrazolium bromide] by the cellular dehydrogenases to a blue formazan soluble only in organic solvents). At the end of the experimental periods, the extraction media was removed and the cells were washed twice with phosphate-buffered saline (PBS, Life Technologies Corporation, Grand Island, NY, USA) and incubated at 37 °C for 3 h in a humidified 5% CO_2_ atmosphere with an MTT solution (1 mg/mL). Finally, the MTT solution was removed and the insoluble formazan was solubilised with DMSO. The optical density (OD) of the final product was measured spectrophotometrically at 550 nm using a microplate reader (FlexStation 3 Microplate Reader, Molecular Devices, San Jose, CA, USA).

(*iii*) Lactate dehydrogenase (LDH) activity

The possible cytotoxicity of the tested precursor materials was also analysed by estimating the cytoplasmic lactate dehydrogenase (LDH) activity released by the cells cultured in the extraction media. LDH is a cytosolic enzyme found in the cytoplasm of all cell types, which is quickly released into the culture medium by non-viable cells with a damaged plasma membrane. The assay was performed at 24 h and 72 h post-culture, by means of an LDH-based In Vitro Toxicology Assay Kit (Sigma-Aldrich Co., St. Louis, MO, USA), according to the manufacturer’s protocol. The absorbance of the final product was measured at 490 nm using a microplate reader (FlexStation 3 Microplate Reader, Molecular Devices, San Jose, CA, USA).

As mentioned above, the ability of the polymeric materials to support cell viability was also investigated through direct contact studies. In this part, the MC3T3-E1 cells were kept in contact with the surfaces of the ABS and PLA samples.

(*iv*) Viability of cells seeded directly on the surface of the tested materials

The MC3T3-E1 cells were seeded directly on the surface of the samples at a density of 10.000 cells/cm^2^ and kept in culture for 24 h and 96 h. The cell proliferation rate was determined with Cell Counting Kit-8 (CCK-8, Promega, Madison, WI, USA) containing a nontoxic dye, WST-8[2-(2-methoxy-4-nitrophenyl)-3-(4-nitrophenyl)-5-(2,4-disulfophenyl)-2H-tetrazolium, monosodium salt], used for continuous cell culturing. The growth medium was removed and replaced with 10% *v*/*v* CCK-8 (containing complete medium), followed by a 2 h incubation period at 37 °C in a humidified 5% CO_2_ atmosphere. After 2 h, the OD was read spectrophotometrically at 450 nm using a microplate reader (FlexStation3 Microplate Reader, Molecular Devices, San Jose, CA, USA).

## 3. Results and Discussion

### 3.1. FTIR-ATR Measurements

**Figure 1:**
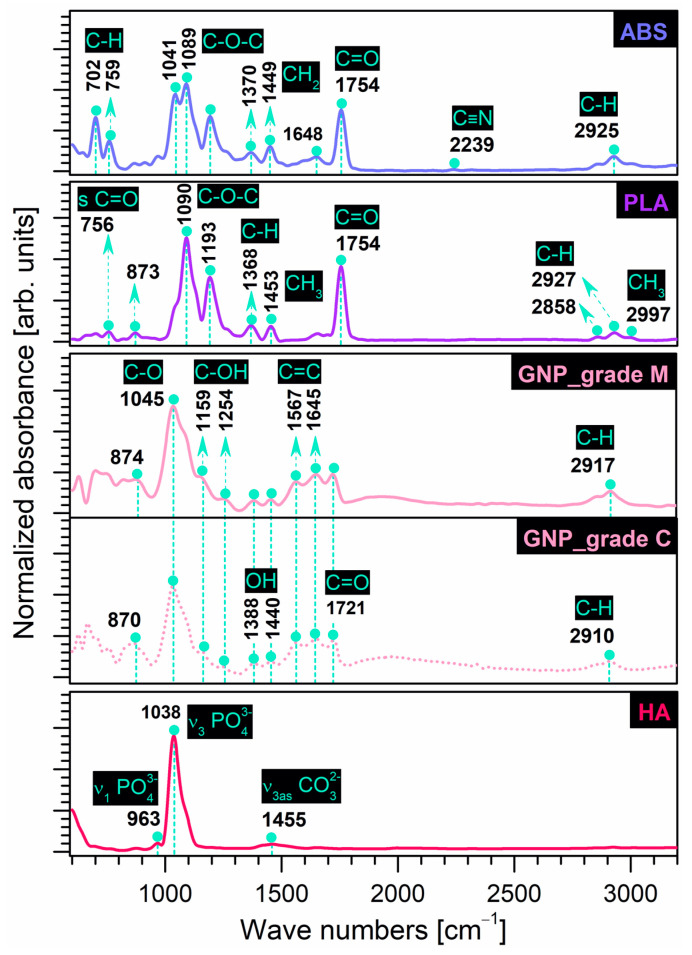
Comparative FT-IR spectra of prime (ABS, PLA) and reinforcement (GNP, HA) precursor materials.

The FT-IR fingerprints of prime and reinforcement precursor materials selected in this study are comparatively presented in Figure 1. Table 1 below offers a complete assignment of IR bands with dedicated references for all samples.

In the case of ***prime materials***, the typical IR bands of the commercial polymers were recorded as follows:

- **ABS**: The bands were attributed to (a) styrene isomers—deformation (out of phase sequence) of C–H groups (702, 759 cm^−1^); (b) acrylonitrile isomers—symmetric stretching of C≡N groups (2239 cm^−1^); and (c) butadiene isomers—asymmetric bending of CH_2_ groups as low-frequency peaks (1370, 1449 cm^−1^), symmetric stretching of C=C (1648 cm^−1^) and C=O (1754 cm^−1^) groups. Additionally, a split-peak corresponding to the symmetric stretching of C–O–C groups and a low-intensity peak associated with the aliphatic symmetric stretching of C–H groups were identified at 1041, 1080 cm^−1^, and 2925 cm^−1^, respectively. However, the emergence of the carbonyl (C=O) stretching band, slightly shifted from its usual position at 1718 cm^−1^ (when identified), could be assigned to an oxidation state of the polymer in the butadiene region, as also found in ref. [7], thus suggesting reduced elasticity features that could interfere with the polymer processing and final mechanical performance.
- **PLA**: The main functional bands were assigned to the C=O groups—symmetric stretching (756, 1754 cm^−1^); C–O–C groups—symmetric (1090 cm^−1^) and asymmetric (1193 cm^−1^) stretching; methyl (CH_3_) groups—symmetric (1453 cm^−1^) and asymmetric (2997 cm-^1^) stretching; and (–CH) groups—symmetric (2858 cm^−1^) and asymmetric (2927 cm^−1^). Additionally, the deformation (bending mode) of the C–H groups and the possible vibration of the C–COO group could be identified as low-intensity peaks at 1368 and 873 cm^−1^, respectively, as also found in refs. [13,25,27].

Similarly, the frequency assignment depicted the characteristic vibrational modes of the selected ***reinforcement materials*** as follows:

- **GNP**: For both types of graphene nanoplatelets (grade C and M), at similar intensities, the characteristic bands were ascribed to C–O groups—symmetric stretching (1045 cm^−1^); C=C groups—aromatic stretching (1567, 1645 cm^−1^); and C=O groups—symmetric stretching (1721 cm^−1^). Comparatively, at very low intensities, the deformation (bending mode) of the hydroxyl (–OH) functionalities and the symmetric (phenolic) stretching of the C–OH groups were identified at 1388, 1440 cm^−1^ and 1159, 1254 cm^−1^, respectively. At wavenumbers slightly shifted to the right for the grade M sample as compared to the grade C sample, the bands for the aromatic deformation of the C–H groups (870, 874 cm^−1^) and the symmetric stretching of CH_2_ groups (2910, 2917 cm^−1^) were also present, as similarly reported in ref. [50]. No additional peaks specific to the hydroxyl group (free or connected) in the >3000 cm^−1^ range were found here, in contrast to other records [37,51,52].
- **HA**: The bands characteristic of the symmetric and asymmetric stretching of orthophosphate tetrahedral units (PO_4_)^3−^ were assigned at 963 cm^−1^ and 1038 cm^−1^, respectively. Compared to pure, highly crystalline commercial HA [53], the spectra of the bovine bone-derived HA also elicited low-intensity bands specific to asymmetric stretching (centred at ~1455 cm^−1^) modes of carbonate groups. Carbonation of calcium phosphates synthesised under normal atmospheric conditions was expected and repeatedly reported in our previous studies [12,15,16]. Moreover, the involved HA is also monophasic and free of any traces of other molecules, confirming the reproducibility of the synthesis method [16,44].

**Table 1:** IR band assignment for prime and reinforcement precursor materials.

ABS	PLA	GNP_C	GNP_M	HA	IR Band Assignment
Wavenumber Positions [cm^−1^]					
702	-	-	-	-	def. (out of phase) of C–H groups in S ^1^ [26,54,55,56] aromatic bending of C–H groups [7]
-	756	-	-	-	sym. stretching of C=O group [21]
759	-	-	-	-	def. (out of phase) of C–H groups in S ^1^ [26,54,55,56] aromatic bending of C–H (=CH) groups [7]
-	-	870	874	-	aromatic deformation of C–H groups [50]
-	873	-	-	-	C–COO group vibrations [13]
-	-	-	-	963	sym. stretching (ν_1_) of (PO_4_)^3−^ groups [3,16,20,44]
-	-	-	-	1038	asym. stretching (ν_3_) of (PO_4_)^3−^ groups [3,16,44]
1041	-	-	-	-	sym. stretching of C–O–C groups [56]
-	-	1045	1045	-	sym. stretching (ν_1_) of C–O groups [52,57,58]
1089	-	-	-	-	sym. stretching of C–O–C groups [56]
-	1090	-	-	-	sym. stretching of C–O–C groups [13,21,59]
-	-	1159	1159	-	sym. (phenolic) stretching (ν_1_) of C–OH groups [50,58]
-	1193	-	-	-	asym. stretching of C–O–C groups [13,21,59]
-	-	1254	1254	-	sym. (phenolic) stretching (ν_1_) of C–OH groups [37,50,58]
-	1368	-	-	-	def. bending of C–H groups [25,27]
1370	-	-	-		asym. bending of CH_2_ groups in B ^3^
-	-	1388 1440	1388 1440	-	def. bending of (–OH) groups [50,58]
1449	-	-	-	-	asym. bending of CH_2_ groups in B ^3^ [7,54,56,60,61]
-	1453	-	-	-	sym. stretching of CH_3_ groups [21,25,27]
-	-	-	-	1455	asym. stretching (ν_3_) of (CO_3_)^2−^ groups [15,16,25]
-	-	1567 1645	1567 1645	-	aromatic stretching of C=C groups [52,57,62]
1648	-	-	-	-	sym. stretching of C=C groups in B ^3^ [7,56,63]
-	-	1721	1721	-	sym. stretching of C=O groups [50,57,58]
1754	-	-	-	-	stretching of C=O group in B ^3^ [7]
-	1754	-	-	-	sym. stretching of C=O groups [20,21,25,27,59,64]
2239	-	-	-	-	sym. stretching of C≡N groups in AN ^2^ [26,56,60,61,63,65]
-	2858	-	-	-	sym. stretching of (–CH) groups [21,25]
-	-	2910	2917	-	sym. stretching of CH_2_ groups [50]
2925	-	-	-	-	aliphatic sym. stretching of C–H groups [7,54,56,60,61,63]
-	2927	-	-	-	asym. stretching of (–CH) groups [13,21,25]
-	2997	-	-	-	asym. stretching of CH_3_ groups [5,13,21,25]

^1^ S—styrene; ^2^ AN—acrylonitrile; ^3^ B—butadiene.

### 3.2. Raman Measurements

**Figure 2:**
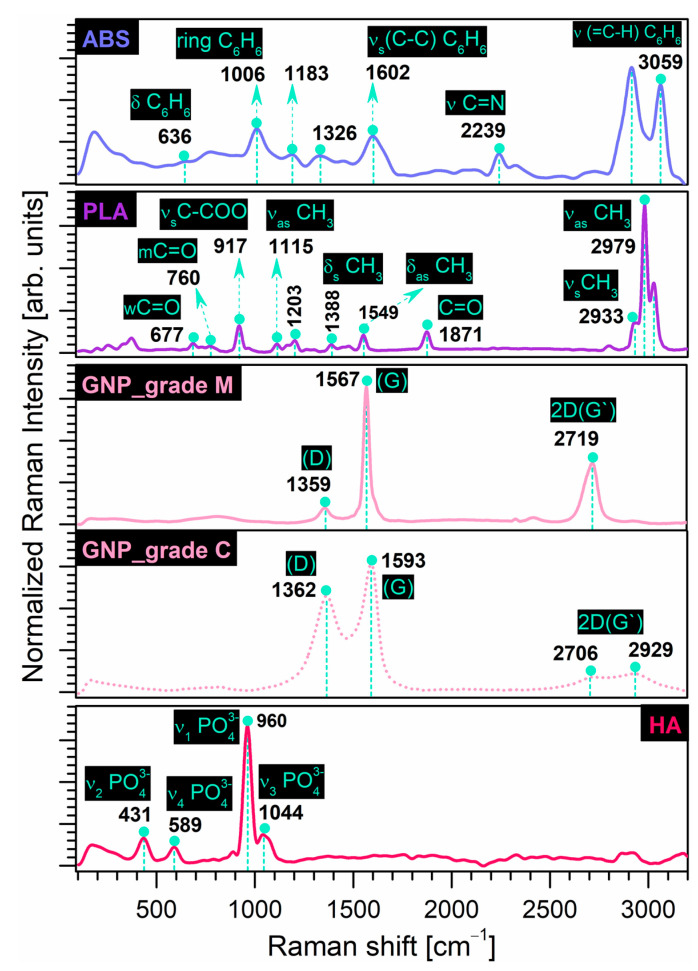
Comparative Raman spectra of prime (ABS, PLA) and reinforcement (GNP, HA) precursor materials.

The Raman fingerprints of the precursor materials investigated in this study are comparatively revealed in Figure 2. Table 2 below offers a complete indexation of all Raman shifts identified for all samples, including dedicated references. The Raman outcomes are in excellent agreement with those disclosed by the FTIR-ATR investigations.

The recorded results for the ***prime materials*** confirmed the identification of all characteristic bands, as presented below. As previously pointed out also in the case of the FT-IR measurements, depending on the manufacturing process/supplier for the commercial materials, the frequencies of all or some of these vibration modes can vary considerably and may be shifted, but still remain in a safe area of values [66,67].

- **ABS**: The main bands were designated to (a) the vibrational modes of the benzene ring in styrene isomers—deformation (in-plane mode) (636 cm^−1^), breathing mode (1006 cm^−1^), shearing (in-plane mode deformation) of the C–H groups (1183 cm^−1^), symmetric stretching of the C–C groups (1602 cm^−1^), and stretching of the (=C–H) groups (3059 cm^−1^) with the highest intensity; (b) the deformation of CH_2_ groups (1326 cm^−1^); and (c) the stretching of C≡N groups (2239 cm^−1^) in acrylonitrile isomers.
- **PLA**: The main vibrational bands corresponded to the C=O groups—weak (677 cm^−1^), moderate (760 cm^−1^) and stretching (1871 cm^−1^) modes; the CH_3_ groups—asymmetric functional mode (1115 cm^−1^), symmetric (1388 cm^−1^), and asymmetric (1549 cm^−1^) deformation of the functional mode, symmetric (2933 cm^−1^) and asymmetric (2979 cm^−1^) stretching modes with the most prominent intensities. In addition, the symmetric stretching of the C–COO groups (917 cm^−1^) and the asymmetric moderate mode of the C–O–C groups (1203 cm^−1^) were confirmed, according to ref. [21].
  In the case of the ***reinforcement materials***, the Raman shifts outlined the following:
- **GNP**: The two sets of intense characteristic peaks for carbon nanostructures were assigned to the D band—specific to disordered/disrupted hexagonal graphitic lattice or internal structural defects [50,57,68] at 1362 cm^−1^ and 1359 cm^−1^ for grade C and M samples, respectively. The G band, generated by the stretching of the functional C–C groups, was also indexed at 1593 cm^−1^ and 1567 cm^−1^ for grade C and M samples, respectively, and is related to the structural disorder [57,68]. It was stated that the broader the G band in the Raman spectra, the higher the oxidation performance of the graphite [50]. As such, given the prominent intensity and narrower G band position, we can assume that for the grade M sample, a significant number of graphitic areas remained unmodified after processing [57,69]. However, for both sample types, the identification of the 2D (G’) band (sensitive to the thickness, number, and stacking of graphene layers in a flake [36,70,71,72]) in the 2700–2930 cm^−1^ range was supported by other studies [70,71,72]. In the case of the GNP_grade M sample, due to the higher diameter surface and thickness of the nanoparticles that interact with the laser beam [70], a well-defined high intensity peak was formed in this area, as expected. It was also reported that the intensity is directly dependent on the number of graphene layers [71].
- **HA**: Only the typical bands of the (PO_4_)^3−^ groups in the HA structure were depicted: the asymmetric (1044 cm^−1^) and symmetric (960 cm^−1^) stretching mode, the bending (431 cm^−1^) mode, and the out-of-plane bending (589 cm^−1^) mode.

**Table 2:** Raman shifts assignment for prime and reinforcement precursor materials.

ABS	PLA	GNP_C	GNP_M	HA	Raman Shift Assignment
Raman Shifts [cm^−1^]					
-	-	-	-	431	bending (ν_2_) of (PO_4_)^3−^ groups [3,13,20,73]
-	-	-	-	589	bending (ν_4_) of (PO_4_)^3−^ groups [3,13,20,73]
636	-	-	-	-	def. (in-plane mode) of benzene ring [66,67,74]
-	677	-	-	-	weak C=O groups [21]
-	760	-	-	-	moderate C=O groups [21]
-	917	-	-	-	sym. stretching of C–COO groups [13,21,64]
-	-	-	-	960	sym. stretching (ν_1_) of (PO_4_)^3−^ groups [3,20,73]
1006	-	-	-	-	breathing of benzene ring [66,67,74]
-	-	-	-	1044	asym. stretching (ν_3_) of (PO_4_)^3−^ groups [3,20,73]
-	1115	-	-	-	asym. functional CH_3_ groups [21]
1183	-	-	-	-	def. shearing (in-plane mode) of C–H groups in benzene ring [66,74]
-	1203	-	-	-	asym. moderate C–O–C groups [21]
1326	-	-	-	-	def. of CH_2_ groups [66]
-	-	1362	1359	-	D (disordered) band [50,57,68,69,75]
-	1388	-	-	-	sym. def. of functional CH_3_ groups [21,76,77]
-	1549	-	-	-	asym. def. of functional CH_3_ groups [21,76,77]
-	-	1593	1567	-	G (graphitic) band [50,57,68,69,75]
1602	-	-	-	-	sym. stretching of C–C groups in the benzene ring in S ^1^ [54,66,67]
-	1871	-	-	-	stretching of C=O groups [13,21,76,77]
2239	-	-	-	-	stretching of C≡N groups in AN ^2^ [54,61,66,67]
-	-	2706	2719	-	2D (G’) band [70,71,72]
-	-	2929	-	-	2D (G’) band [70,71,72]
-	2933	-	-	-	sym. stretching of CH_3_ groups [13,21,77]
-	2979	-	-	-	asym. stretching of CH_3_ groups [13,21,77]
3059	-	-	-	-	stretching of (=C–H) groups in benzene ring [66,67,74]

^1^ S—styrene; ^2^ AN—acrylonitrile.

### 3.3. Morphology Evaluation

**Figure 3:**
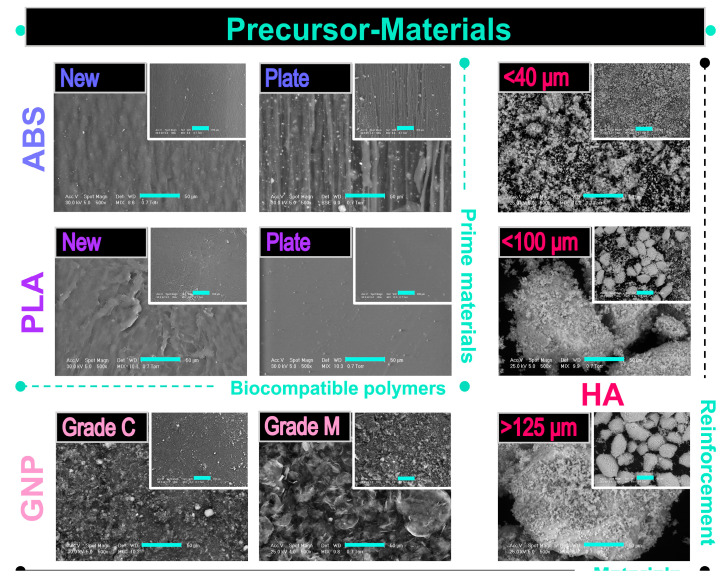
Representative SEM images of prime (ABS, PLA) and reinforcement (GNP, HA) precursor materials acquired at two magnifications; scale bar: 50 µm (main image) and 200 µm (inset image).

The morphological evaluation results are comparatively exposed in Figure 3 for both categories of selected precursor materials.

The ***prime materials*** were investigated in two physical shapes, i.e., new, as-received (initial) materials and prepared plates from thin lamellas.

- **ABS**: Regardless of the physical state, the ABS surface outlined the presence of a continuous and homogenous matrix (also known as the SAN matrix) and some light-white sub-micrometric inclusions corresponding to the butadiene-specific particle content, as mentioned in refs. [61,65]. The microstructure of the new material depicted a slightly rough and uneven distribution of the two phases at a higher magnification. Moreover, the ABS in the form of a thin plate conversed to a morphology of rubbery-like lines and grooves after flattening. Implicitly, in this state, a more prominent roughness and irregular disposal of the content materials occurred.
- **PLA**: The initial PLA granules mainly displayed a wrinkled surface with few pores of sub-micrometric diameters. In contrast, the processing into a plate shape acted as an adjuvant for uniform surface smoothing and complete reduction of the pores/voids.

Concerning the morphological features of the ***reinforcement materials***, the following observations were drawn:

- **GNP**: The microstructure was clearly influenced by the GNP nanoplatelet dimensions: the grade C samples demonstrated an accentuated tendency to form clusters/irregular aggregates with a width of a few micrometres, which appeared mostly as particulates, rather than as platelets structures, which is consistent with other reported findings [36,38]. This predilection of the GNP_grade C graphene is well-known and attributed to the strong van der Waals interactions [50]. Comparatively, the grade M samples exposed a unitary flake-like microstructure with well-separated and layered nanoplatelets of variable sizes and orientations [58,62,71].
- **HA**: The coarse surface and polyhedral form of the ceramic particles were conserved for all dimensional sorts. However, if the particles size was in the <40 μm range, they tended to conglomerate into larger micrometric aggregates. Additionally, as a direct consequence of the synthesis technology [45], the HA particles acquired a microporous surface, which is considered to be beneficial for a more intimate contact and adhesion to other materials used as matrix for composite filaments (e.g., polymeric materials) [43]. When addressed differently, this feature of the ceramic particles is sometimes challenging and rather difficult to achieve [5,27].

### 3.4. In Vitro Pre-Osteoblast Behaviour

**Figure 4:**
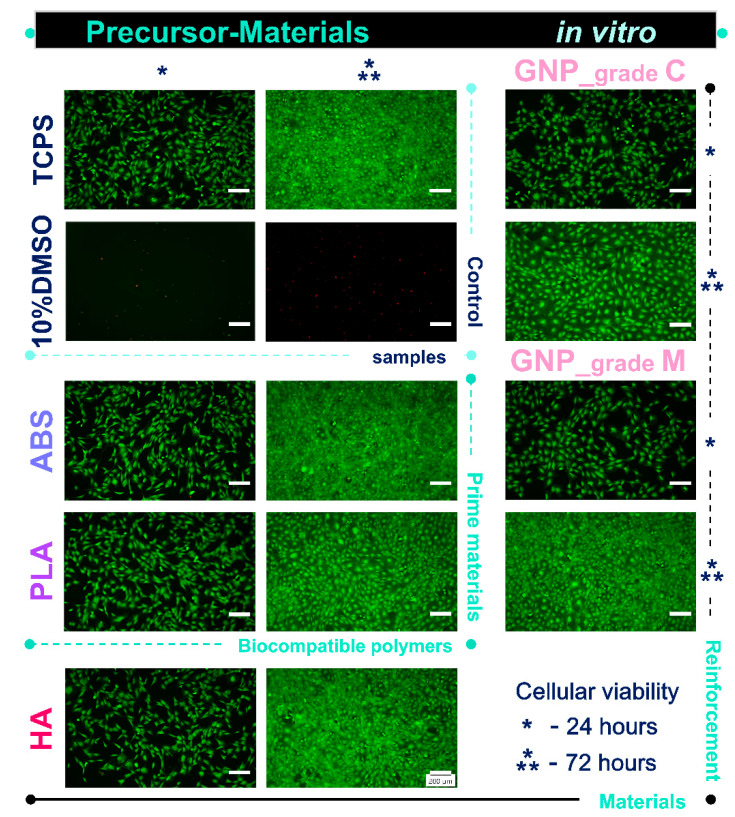
Fluorescence micrographs of the MC3T3-E1 pre-osteoblasts grown in the extraction media of precursor materials for 24 h and 72 h. The Live/Dead assay revealed the exclusive presence of green labelled viable cells in case of all analysed samples, except the positive cytotoxicity control where red fluorescent dead cells were noticed. Scale bar: 200 µm.

The Live/Dead assay was conducted in order to investigate the cytocompatibility of the precursor materials. Thus, Figure 4 indicated that after 24 h of culture, the pre-osteoblasts were viable (green fluorescence) and healthy, with no significant differences between the cells grown in various extraction media collected from the analysed samples. Furthermore, after 72 h of culture, the number of viable cells increased substantially for all analysed extraction media, suggesting that the tested materials exhibited, on different levels, the ability to support cellular survival and proliferation. It was noticed that of all the analysed materials, the extraction media collected from the GNP_grade C precursor indicated the lowest cell density after 72 h of culture. These results confirmed the previsioned influence of GNP size upon cell viability [41,42].

**Figure 5:**
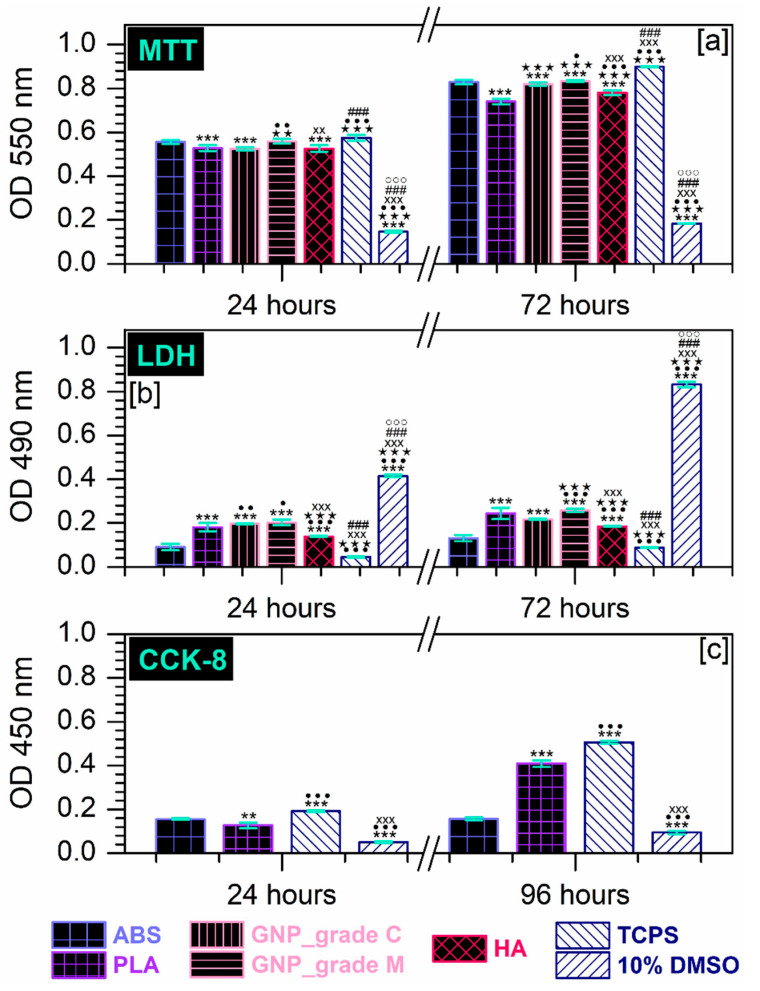
(**a**) Extract effect on the degree of survival and proliferation potential of MC3T3-E1 pre-osteoblasts at 24 h and 72 h after seeding, as assessed by the MTT assay (*** p < 0.001 vs. *ABS*; ^★★★^ *p* < 0.001, ^★★^ *p* < 0.01 vs. *PLA*; ^•••^ *p* < 0.001, ^••^ *p* < 0.01, ^•^ *p* < 0.05 vs. *GNP_grade C*; ^xxx^ *p* < 0.001, ^xx^ *p* < 0.01 vs. *GNP_grade M*; ^###^ *p* < 0.001 vs. *HA*; ^°°°^ *p* < 0.001 vs. *TCPS*). (**b**) Analysis of LDH activity released in the culture medium by MC3T3-E1 cells incubated for 24 h and 72 h in the extraction media of the precursor materials (*** *p* < 0.001 vs. *ABS*; ^•••^ *p* < 0.001, ^••^ *p* < 0.01,*^•^ p* < 0.05 VS. *PLA*; ^★★★^ *p* < 0.001 vs. *GNP_grade C*; ^xxx^ *p* < 0.001 vs. *GNP_grade M*; ^###^ *p* < 0.001 vs. *HA*; ^°°°^ *p* < 0.001 vs. *TCPS*). (**c**) Survival/proliferation rates of MC3T3-E1 cells seeded directly on the surface of the prime materials after 24 h and 96 h of incubation and evaluated through the CCK-8 assay (*** *p* < 0.001, ** *p* < 0.01 vs. *ABS*; ^•••^ *p* < 0.001 vs. *PLA*; ^xxx^ *p* < 0.001 vs. *TCPS*).

Moreover, the morphological observations are consistent with the results of the MTT assay presented in Figure 5a. As displayed, the tested extraction media were capable of providing a suitable environment for cell proliferation, which led to an elevated number of metabolically active viable cells with the incubation time. However, the proliferation rates, reflected by the O.D. values, showed significant differences between the MC3T3-E1 pre-osteoblasts grown in different extraction media. Thus, after 72 h of culture, the highest proliferation rate was recorded for the cells grown in the extraction media collected from the ABS and GNP_grade M samples, while the lowest O.D. values were recorded for the pre-osteoblasts maintained in the PLA extract.

Additionally, the release of LDH into the culture media was measured as an index of cellular death (the possible harmful effect exerted by the precursor materials on the cell viability). As depicted from Figure 5b, the MC3T3-E1 cells displayed significantly comparable survival rates between the analysed samples, regardless of the experimental time points. The time-dependent LDH activity profiles revealed that after 72 h of culture, the lowest enzymatic activity was recorded in the extraction media harvested from the HA and GNP_grade C samples, while the cells seeded in the ABS and GNP_grade M extracts presented a higher membrane integrity loss coupled with a subsequent increase in the LDH extracellular activity.

Altogether, the cytocompatibility tests performed according to ISO 10993-5 standards (ref. [49]) on the MC3T3-E1 pre-osteoblasts, demonstrated that the investigated extraction media exerted a sparse cytotoxic effect. Therefore, an additional in vitro investigation was carried out to allow a more precise selection of the polymeric precursors that will be used for the development of composite materials in future studies.

To this end, the biological performance of the prime-materials substrates was further evaluated via the CCK-8 assay at 24 h and 96 h post-seeding in order to determine their potential to sustain the MC3T3-E1 pre-osteoblasts viability. The in vitro studies were carried out through direct contact between the pre-osteoblasts and the PLA and ABS plate substrates, and the collected results are presented in Figure 5c. Here, the MC3T3-E1 cells exhibited a time-dependent progressive increase in the O.D. values that altogether displayed significant differences between the cells grown on the surface of the tested materials. In consequence, at 24 h after plating the cells grown on the ABS sample, higher O.D. values were recorded compared with the PLA sample. This trend reversed at 72 h, where the number of metabolically active viable cells decreased as follows: TCPS > PLA >ABS > 10% DMSO.

In light of this, the obtained results indicated that between the two tested surfaces, the ABS precursor elicited a potential cytotoxic effect when cells were seeded directly on its surface, a behaviour which was more pronounced at 96 h post-culture. In this regard, a possible explanation could be represented by the samples surface topography, a property that plays an important role in the attachment of cells. For example, Herath et al. [78] investigated the response of a human osteosarcoma cell line to zirconia ceramic surfaces and observed that, initially, the more abraded surfaces were capable of providing a better support for cell attachment and cell spreading. Over time, this behaviour changed, and the highest rate of cell proliferation and expression of phenotypic markers were recorded on polished surfaces, suggesting that the uneven surfaces lost their ability to support cell attachment; thus, lower O.D. values were recorded due to the reduced number of cells. Similarly, Schmelzer et al. [79] observed that after 48 h in culture, the attachment rates of bone marrow mesenchymal stromal cells (BM-MSCs) to PLA were higher compared to the ABS support and the control samples. Considering this, our O.D. values for the ABS sample at 24 h post-culture could be due to an increased initial cell attachment. Moreover, given the morphology of the ABS plates described above (Figure 3), considered favourable for cell viability and proliferation [11], the cells death recorded after longer periods of incubation is strictly a chemical bound process.

Based on the results of the in vitro cytocompatibility tests as a whole, we can establish the necessity to exclude the GNP_grade C and ABS precursor materials from the technological protocol for developing composite filaments in close regard to the biological requirements for biomedical applications.

## 4. Conclusions

In this work, two categories of precursor materials were comprehensively investigated by physico-chemical, morphological, and in vitro biological assays, targeting an adequate selection route for the materials intended for the future development of composite filaments used in FDM technology. We successfully eliminated the precursor materials with inadequate features for the envisioned biomedical applications.

As such, the complementary FTIR-ATR and Raman investigations exposed only bands assigned to the characteristic vibrational modes, free of any traces, impurities, or other molecules/compounds, in the case of both the prime (ABS and PLA) and the reinforcement materials (GNP—grade C vs. grade M clearly delineated, and bovine bone derived HA).

Morphological evaluation of the prime materials for the new ABS sample revealed a typical combination of the SAN matrix and white micrometric particles from the butadiene content. In contrast, the initial PLA granules presented wrinkled surfaces with few sub-micrometric pores. However, when modelled into thin plates, the polymeric materials outlined dissimilar microstructures: a linear disposal of grooves and an overall increased roughness for ABS versus a very smooth surface for PLA.

GNP sample examination demonstrated an accentuated tendency to form clusters in the form of particulates in the case of grade C type, while the grade M type retained the well-separated flake-like appearance of the graphene nanoplatelets. Regarding the three types of HA particles, their features were, once again, preserved after the full-conversion protocol from bovine bones, thus confirming the reproducibility of this synthesis route.

The final biological assessment enabled the exclusion of only one material from each category: the ABS and GNP_grade C samples, due to their pronounced cytotoxic effect. The direct contact tests performed only on the polymeric plates further confirmed the significant cytotoxic potential of the ABS material as compared to the PLA material. Thus, in our next study, we will proceed with the development of composite filaments based on PLA (as the polymeric matrix), reinforced with both GNP_grade M and HA, in close regard to the requirements for biomedical applications in the orthopaedic and dental fields.

## Data Availability

Data is contained within the article.
